# Draft Genome Sequences of Two Clostridium algidicarnis Strains Isolated from Meat Juice Samples of Chilled Vacuum-Packed Lamb Meat

**DOI:** 10.1128/MRA.00983-20

**Published:** 2020-11-05

**Authors:** Joseph Wambui, Nicole Cernela, Marc J. A. Stevens, Giovanni Ghielmetti, Roger Stephan

**Affiliations:** a Institute for Food Safety and Hygiene, Vetsuisse Faculty, University of Zurich, Zurich, Switzerland; University of Rochester School of Medicine and Dentistry

## Abstract

Clostridium algidicarnis causes blown-pack spoilage of vacuum-packed meat. Here, we report the draft genome sequences of *C. algidicarnis* strains CM002 and CM003, isolated from unspoiled chilled vacuum-packed lamb. The genome sequences of CM002 and CM003 comprise 2,950,326 and 2,870,995 bp, respectively, and each have a GC content of 30.1%.

## ANNOUNCEMENT

Clostridium algidicarnis belongs to the group of psychrophilic and psychrotrophic clostridia causing blown-pack spoilage (BPS) in chilled vacuum-packed meat (1). Despite the role of this species in BPS, genomic studies of C. algidicarnis are limited. We report the draft genome sequences of the two nonhemolytic *C. algidicarnis* strains CM002 and CM003, isolated from meat juice samples from chilled vacuum-packed lamb (2).

The strains were isolated from two different meat juice samples following a multistep sample preparation process involving ethanol (50% [vol/vol]; 1 h; 30°C) and lysozyme (4 mg/ml; 30 min; 37°C) treatment followed by anaerobic enrichment at 4°C in prereduced peptone-yeast-glucose-starch medium for 3 weeks. A loopful of the respective enriched sample was plated onto Columbia agar supplemented with 5% defibrinated sheep blood (CBA) and incubated further anaerobically at 4°C for 3 weeks. Single colonies were incubated anaerobically on CBA at 4°C for 2 weeks for purification. Anaerobic conditions were generated in anaerobic jars using Thermo Scientific Oxoid AnaeroGen sachets.

A DNA blood and tissue kit (Qiagen, Hombrechtikon, Switzerland) was used to extract genomic DNA from a single colony purified on the CBA. Illumina Nextera DNA Flex chemistry and a MiniSeq sequencer (Illumina, San Diego, CA, USA) were used for the preparation and sequencing of transposon-based libraries, respectively. The sequence reads were 1,335,527 and 755,507 bp for CM002 and CM003, respectively. For both genome sequences, paired-end sequence outputs of 150 to 300 bp were produced. Quality control was carried out using FastQC 0.11.7 (3) before assembly using the SPAdes 3.0-based software (4) Shovill 1.0.9 (https://github.com/tseemann/shovill). Contigs of >500 bp were selected for assembly. The assembled genomes of CM002 and CM003 comprise 2,950,326 and 2,870,995 bp, respectively. Each genome has a GC content of 30.1%. The number of contigs, genome coverage, contig *N*_50_ value, and contig *L*_50_ value for CM002 are 54, 45×, 185,888 bp, and 6, respectively, while for CM003, they are 56, 65×, 182,926 bp, and 7, respectively. CheckM (5) was used to assess the quality of both genomes. Default parameters were used for all software and Web servers.

Strain identification was carried out *in silico* using 16S-based identification tools (5, 6) and whole-genome sequence-based digital DNA-DNA hybridization (dDDH) (7) and average nucleotide identity (ANI) (8) against *C. algidicarnis* strains B3^T^ (GenBank accession no. JNLN00000000) and DSM 15099 (PTIS00000000). Comparative dDDH analyses for subspecies delimitation resulted in values ranging between 48.7 and 65.8%, which are below the 79% threshold (7), indicating that strains CM002 and CM003 are phylogenetically distinct.

Gene annotation and prediction were carried out using the NCBI Prokaryotic Genome Annotation Pipeline (PGAP) (9) and Rapid Annotations using Subsystems Technology (RAST) (10). Genes encoding proteolytic, lipolytic, and saccharolytic proteins were predicted, thus providing an insight into the metabolic features of these strains that might be involved in BPS.

### Data availability.

The whole-genome shotgun project for CM002 and CM003 has been deposited at DDBJ/ENA/GenBank under the accession no. JACKWX000000000 and JACKWW000000000, respectively. The versions described in this paper are JACKWX010000000 and JACKWW010000000, respectively. The raw sequencing reads have been deposited in the SRA under the accession no. SRX8947912 and SRX8947913, respectively.
